# Cytomegalovirus in the transplant setting: Where are we now and what happens next? A report from the International CMV Symposium 2021

**DOI:** 10.1111/tid.13977

**Published:** 2022-11-11

**Authors:** Camille N. Kotton, Julián Torre‐Cisneros, The International CMV Symposium Faculty, José Maria Aguado, Sophie Alain, Fausto Baldanti, Gabriele Baumann, Udo Boeken, Maria de la Calle, Javier Carbone, Fabio Ciceri, Patrizia Comoli, Lionel Couzi, Lara Danziger‐Isakov, Mario Fernández‐Ruiz, Corrado Girmenia, Paolo Antonio Grossi, Hans H. Hirsch, Atul Humar, Nassim Kamar, Camille Kotton, Per Ljungman, Michele Malagola, Estefania Mira, Nicolas Mueller, Martina Sester, Chieh‐Lin Jerry Teng, Julian Torre‐Cisneros, Piedad Ussetti, Glen Westall, Dana Wolf, Marty Zamora

**Affiliations:** ^1^ Transplant and Immunocompromised Host Infectious Diseases, Infectious Diseases Division, Massachusetts General Hospital Harvard Medical School Boston Massachusetts USA; ^2^ Maimónides Institute for Biomedical Research of Cordoba (IMIBIC)/Reina Sofía University Hospital/University of Cordoba (UCO) Cordoba Spain; ^3^ CIBERINFEC, ISCIII ‐ CIBER de Enfermedades Infecciosas Instituto de Salud Carlos III Madrid Spain; ^4^ University Hospital 12 de Octubre CIBERINFEC, ISCIII ‐ CIBER de Enfermedades Infecciosas, Instituto de Salud Carlos III Madrid Spain; ^5^ French References Center for Herpes Viruses Microbiology Department CHU‐Limoges Limoges France; ^6^ Università di Pavia, Fondazione IRCCS Policlinico San Matteo Pavia Italy; ^7^ Landeskrankenhaus Steyr Steyr Austria; ^8^ Department of Cardiac Surgery Medical Faculty and University Hospital Düsseldorf Heinrich‐Heine‐University Düsseldorf Germany; ^9^ La Paz Hospital Madrid Spain; ^10^ Hospital General Universitario Gregorio Marañón Madrid Spain; ^11^ IRCCS San Raffaele Scientific Institute University Vita‐Salute San Raffaele Milan Italy; ^12^ Cell Factory and Center for Advanced Therapies and Pediatric Hematology/Oncology Fondazione IRCCS Policlinico San Matteo Pavia Italy; ^13^ Department of Nephrology Transplantation, Dialysis and Apheresis, CHU Bordeaux CNRS‐UMR 5164 ImmunoConcEpT, Bordeaux University Bordeaux France; ^14^ Cincinnati Children's Hospital Medical Center and University of Cincinnati Cincinnati USA; ^15^ University Hospital 12 de Octubre Madrid Spain; ^16^ AOU Policlinico Umberto I Rome Italy; ^17^ Università degli Studi dell'Insubria – ASST SetteLaghi Varese Italy; ^18^ University Hospital Basel Basel Switzerland; ^19^ University of Toronto Toronto Canada; ^20^ Toulouse University Hospital Toulouse France; ^21^ Massachusetts General Hospital Boston USA; ^22^ Karolinska Hospital and Karolinska Institute Stockholm Sweden; ^23^ University of Brescia Brescia Italy; ^24^ Reina Sofía University Hospital Cordoba Spain; ^25^ Universitäts Spital Zürich Zürich Switzerland; ^26^ Universität des Saarlandes Homburg Germany; ^27^ Taichung Veterans General Hospital Taichung Taiwan; ^28^ Reina Sofía University Hospital Cordoba Spain; ^29^ Hospital Puerta de Hierro Majadahonda Madrid Spain; ^30^ Alfred Hospital Melboune Australia; ^31^ Hadassah University Medical Center Jerusalem Israel; ^32^ University of Colorado at Denver Anschutz Medical Center Colorado USA

**Keywords:** cellular therapy, cytomegalovirus immunoglobulin (CMVIG), hematopoietic stem cell transplant, prophylaxis, solid organ transplant, treatment, vaccine

## Abstract

The CMV Symposium in September 2021 was an international conference dedicated to cytomegalovirus (CMV) infection after solid organ or hematopoietic stem cell transplantation. This review provides an overview of the presentations given by the expert faculty, supplemented with educational clinical cases. Topics discussed include CMV epidemiology and diagnosis, the burden of CMV infection and disease, CMV‐specific immunity and management of CMV in transplant settings. Major advances in the prevention and treatment of CMV in the past decade and increased understanding of CMV immunity have led to improved patient outcomes. In the future, management algorithms may be individualized based on the transplant recipient's immune profile, which will mark the start of a new era for patients with CMV.

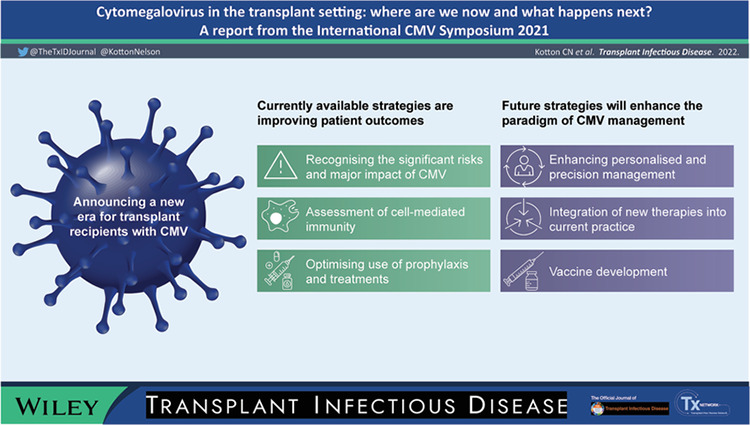

**Abbreviations**
AD: antigenic domain
APC: antigen presenting cell
CD: cluster of differentiation
CMI: cell‐mediated immunity
CMV: cytomegalovirus
CMVIG: cytomegalovirus immunoglobulin
D: donor
ELISA: enzyme‐linked immunosorbent assay
gB: glycoprotein B
GvHD: graft‐versus‐host disease
HGG: hypogammaglobulinemia
HLA: human leukocyte antigen
HSCT: hematopoietic stem cell transplant
IFN: interferon
IG: immunoglobulin
MHC: major histocompatibility complex
NK: natural killer cell
QNAT: quantitative nuclear assay test
R: recipient
RIA: radioimmunoassay
SOT: solid organ transplant
TAP: transporter associated with antigen processing
VST: virus‐specific T cell

## 1 INTRODUCTION TO THE MEETING AND CYTOMEGALOVIRUS

### 1.1 Meeting overview

There have been major advances in the prevention and treatment of cytomegalovirus (CMV) in the past decade, which has resulted in a major impact given the significant burden of CMV in both hematopoietic stem cell transplant (HSCT) and solid organ transplant (SOT) settings. The International CMV Symposium took place in Amsterdam, The Netherlands in September 2021 and provided a valuable forum for delegates, attending in person or virtually, to share insights and ideas about the future of CMV management. The main aim of the meeting was to discuss best CMV prevention methods and how to improve treatment outcomes in transplant settings. An international faculty led the discussions during the meeting and encouraged debate and collaboration. This article will review the presentations and discussions at this meeting, covering the topics of burden of CMV infection and disease, the consequences of CMV infection in transplant settings, and the current and future management of CMV.

### 1.2 CMV and the importance of immune evasion in the transplant setting

CMV is a unique virus, termed “*confoundomegalovirus*” by Hans H. Hirsch because of its ability to persist in long‐lived cells of the myeloid lineage and to reactivate and to aggravate clinical conditions linked to innate and adaptive immune activation. The strong host immune response elicited by CMV is usually able to curtail viral replication effectively, but the virus also uses several immune‐evasion strategies, permitting lifelong latency. In healthy individuals a dynamic balance is maintained between host control and CMV replication.^1^ However, CMV becomes of clinical relevance when there is significant inflammation, stress or the need to use drugs for stabilizing critically ill‐patients (i.e., those acting via the cyclic adenosine monophosphate/protein kinase A pathways) since they promote CMV immediate early gene expression and hence viral replication in CMV‐harboring immune cells.^2^ CMV reactivation is regularly controlled by cytotoxic CD8 T‐cells; however, in patients treated with immunosuppressants, such as in the transplant setting, both the failing immune control and the stimulated CMV replication shift the net balance toward clinically significant reactivation. These events can occur locally in the affected organ compartment and may go undetected by molecular viral load testing in the blood. Thereby CMV contributes to direct and indirect effects locally and systemically, both of which may be altered by antiviral drugs given for prophylaxis or as preemptive management.

Martina Sester described that the classical major histocompatibility complex (MHC) antigen presentation pathway is targeted by CMV at multiple conserved processes. In CD8+ cells specifically, viral CMV‐produced US2 and US11 proteins dislocate MHC class I heavy chain molecules from the endoplasmic reticulum to the cytoplasm, exposing the host proteins to proteasomal degradation, preventing viral presentation and detection by the host immune cells.^3^ Viral US10 also plays a pivotal role in immune evasion, delaying maturation of MHC class I molecules and degradation of human leukocyte antigen (HLA)‐G molecules (a natural killer cell inhibitory receptor ligand). Host peptide loading mechanisms are targeted via viral US6 action on the transporter associated with antigen processing protein (TAP) and US3 inhibition of tapasin, preventing MHC molecule translocation to the endoplasmic reticulum and cell surface expression. These evasion methods culminate in a large population of CMV‐specific T cells being required to control CMV. In transplant patients, this is reflected by a large number of CMV‐specific T cells being detectable.^4^

### 1.3 Risk of CMV in transplant settings

Per Ljungman reminded delegates that a CMV‐positive recipient coming in for HSCT has a lower chance of survival than CMV‐negative individuals when other factors (i.e., age) are taken into consideration.^5^, ^6^ This impact on survival can either be a direct consequence of CMV disease (i.e., end‐organ disease or systemic viral infection) or an indirect outcome resulting from an increased risk of opportunistic bacterial, viral or fungal infection, graft rejection, thrombotic events, or cardiovascular disease.^7^, ^8^, ^9^, ^10^

Poor survival is not only associated with CMV status in the recipient, as using stem cells from a CMV‐positive donor in a CMV‐negative recipient also increases the risk of mortality.^11^ Per Ljungman set the tone of the symposium early by engaging the delegates in discussion during the first presentation of the meeting. He asked where CMV status would fit in the hierarchy of risk when transplanting cells from a CMV‐negative donor into a CMV‐positive recipient. He suggested that HLA matching would be at the top of the list, but queried whether the importance of CMV status was as well established. Given the potentially high burden of CMV in HSCT recipients, it is important to remember that CMV can be prevented either by prophylaxis (using antiviral therapies in either all patients or in subgroups deemed at high risk of disease) or by use of pre‐emptive treatment (early diagnosis of viral replication and prescription of antiviral therapy before the appearance of clinical disease).^12^

When discussing effective management of CMV in HSCT and SOT recipients, an additional consideration is the need to balance use of immunosuppressants with risk of infection and disease progression. Nicolas Mueller explained that, in the HSCT setting, patients on immunosuppressants post‐transplant were most at risk of viral infection, with herpes viruses (although not solely CMV) being the most prevalent.^13^ In contrast, SOT recipients are most likely to have bacterial infections with viral infections occurring infrequently.^14^ The increasing use of biological agents for a range of diseases that also suppress the immune system means that transplant recipients may have a risk profile for CMV infection independent of the risk associated with transplant. Nicolas Mueller concluded by advocating for a thorough evaluation, including a detailed history and screening for viruses, prior to initiating infection prevention strategies, in order to optimize patient outcomes.

### 1.4 Testing for CMV in transplant settings

It is clear establishing CMV status in both recipient and donor is pivotal to understanding the risk of disease development. Gabriele Baumann described how CMV can be detected, the pros and cons of different methodologies and also introduced the concept of monitoring cell‐mediated immunity (CMI) to guide disease management. Key points about testing for CMV and CMI discussed by Gabriele Baumann are summarized in Table 1.^12^, ^15^

**TABLE 1:** When and how to test for CMV^12^, ^15^

	**Virus detection**	**Assess and monitor immune response**
Rationale for testing	To predict risk of CMV after transplant and to guide decisions on management. Post‐transplant, decline in viral load indicates clinical resolution of CMV infection while a rise/minimal decline suggests refractory or drug‐resistant CMV	To assess evidence of prior CMV infection To predict risk of CMV infection post‐transplant and risk of CMV infection at the end of prophylaxis To determine the need for secondary prophylaxis or predict risk of CMV relapse
What to test?	*Virus*: viral proteins (antigens), nucleic acids	*Antibodies*: serological assays (ELISA, RIA) *CMI*: cytokine release, specifically IFNγ from cells following stimulation with CMV‐specific antigens
Who to test?	Recipients	Donors (antibodies) Recipients (antibodies, CMI)
What tests are available	*Viral proteins*: antigenemia (detects pp65 antigen), Shell vial assay, histopathology *CMV nucleic acid*: QNAT *Cytopathic effect*: cell culture of fibroblasts, histopathology	*Antibodies*: serological assays (ELISA, RIA) *CMI*: EliSpot (measures IFNγ, targets CD4+/CD8+), ELISA (measures IFNγ, targets CD8+), flow cytometry (measures IFNγ and CD69, targets CD4+/CD8+)
When to test?	*QNAT*: test weekly post‐transplant for the first 12 weeks and as frequently as weekly thereafter to monitor response to antiviral treatment. This is preferred over antigenemia as it is more standardized. QNAT can also be used to test for active disease and monitor treatment response	Pretransplant and post‐transplant
Considerations	WHO International Standard for human CMV has reduced the variability of CMV DNA results reported on individual samples, but clinically relevant variability persists preventing meaningful inter‐assay comparisons	*Serology* IgM is not recommended routinely due to false positivity Use for CMV diagnosis after transplant is limited *CMI* Thresholds are currently poorly defined for positive and negative results Variability in CMV antigen stimulation protocols exists Most of these tests are currently only for research use

Abbreviations: CMI, cell‐mediated immunity; CMV, cytomegalovirus; ELISA, enzyme‐linked immunosorbent assay; IFN, interferon; QNAT, quantitative nuclear assay test; RIA, radioimmunoassay.

## 2 CMV IN THE TRANSPLANT SETTING—WHERE ARE WE NOW?

### 2.1 Is CMV still a major problem in HSCT?

Numerous risk factors have been identified that increase the chance of a recipient developing CMV disease following HSCT. The recipient's and donor's serological status and the graft type were just a few of those discussed initially by Per Ljungman and later in the meeting by Chieh‐Lin Jerry Teng.

Given that various management options are now available, it is important to assess the risk for CMV disease in order to guide management. Insights on risk in untreated individuals can be estimated by looking at the control arms of HSCT clinical trials or by considering real‐world data. Per Ljungman showed clinical trial data estimating that the incidence of CMV disease in HSCT was approximately 3% at day 100 after transplant,^16^ but higher rates have been reported in real‐world settings. For example, 10.5% of patients had CMV disease after HSCT in the first year after transplant in a retrospective 12‐year study by Green and colleagues.^17^ Given the high rates of CMV disease, it is therefore important to identify strategies to control CMV disease in the HSCT setting. These strategies should focus on prevention of infection and reduction of viral replication, with Per Ljungman stressing that treating established disease represents a failure of strategy. When focusing on prevention, measures such as careful donor selection, use of safe blood products and antiviral prophylaxis are appropriate and effective strategies.

Fausto Baldanti highlighted the specific needs in immunocompromised patients in the HSCT setting, with CMV risks being particularly high in seropositive recipients (i.e., harboring latent CMV infection).^18^ In the immunocompromised population, the reactivation of CMV following treatment promotes selection of drug‐resistant CMV strains^19^ due to reduced immune competence, and Fausto Baldanti proposed that T‐cell assays against CMV such as flow cytometry and EliSpot may be appropriate approaches to determine T‐cell immune competence.^20^, ^21^, ^22^ Determining the best threshold for immune markers to allow accurate prediction of CMV risk and to inform subsequent management will be a key factor in optimal utilization of these tests. Centers without access to virus‐specific tests can still assess immune competence by monitoring T‐cell count, as an absence of CD4/CD8‐positive T cells indicates the need for further investigation.

### 2.2 Management of CMV in the HSCT setting

There is a clear role for using antiviral drugs prophylactically in individuals at risk of CMV disease.^23^ Per Ljungman highlighted the need to assess the evidence carefully. Early studies demonstrated some effectiveness with acyclovir and valaciclovir, and while ganciclovir and foscarnet are effective, there are concerns relating to toxicity with these agents in HSCT.^24^, ^25^, ^26^ However, he explained that letermovir has been shown to result in a significant reduction in clinically significant CMV infection in HSCT and also in all‐cause mortality.^5^, ^27^ All‐cause mortality was an exploratory endpoint in the letermovir prophylaxis study in HSCT recipients, and a significant difference between letermovir and placebo was seen at Week 24 (10.2% and 15.9%, respectively, *p* = .03).^27^ In a subsequent post hoc analysis of the data set, the hazard ratio for all‐cause mortality was .58 (95% confidence interval .35–.98; *p* = .04) at Week 24.^28^ Additional data from real‐world studies are anticipated.

Corrado Girmenia provided an overview of the significant changes to the management of CMV infection in HSCT recipients that have been adopted since the introduction of letermovir. In the letermovir era, the CMV serostatus of the recipient not only determines donor choice and defines transplant risk but may also indicate the need for letermovir prophylaxis. Several clinical studies have shown that letermovir is able to reduce the rate of clinically significant CMV infection in high‐ and low‐risk CMV‐seropositive HSCT recipients,^27^, ^28^ and some real‐world data are now available to support these findings.^29^ Despite these promising results, the impact of letermovir prophylaxis on mortality is less clear, and more long‐term data are required.^28^

Recent international guidelines give a high level of recommendation for letermovir prophylaxis in CMV‐seropositive recipients following HSCT.^24^ However, there are still several open issues in the management of CMV after HSCT in the letermovir era including the management of low‐level CMV DNAemia during letermovir prophylaxis.^30^ These increases in CMV DNA are often referred to as “blips.”

Blips during letermovir therapy was a topic addressed by Michele Malagola who described how approximately 30% of HSCT recipients will experience increased levels of CMV DNA during treatment.^30^ Where antiviral agents (e.g., ganciclovir) that target DNA polymerases prevent the production of CMV DNA and release of infectious virions into the blood, letermovir acts by inhibiting terminases, which are important for the production of virions but do not inhibit DNA synthesis. The blips observed are non‐infectious CMV DNA fragments released into the blood resulting in CMV DNAemia blips and are an expression of abortive infection.^30^ Corrado Girmenia suggested that using early shell vial culture or DNAase tests can help when deciding on whether to continue letermovir therapy (if tests are negative) or start antiviral therapy with ganciclovir, valganciclovir or foscarnet or immunotherapy (if test is positive).^31^ Where tests are not available, then it is necessary to wait to see if blips continue to occur before making management decisions as treatment may not always be necessary.

There was consensus that letermovir has transformed the way that CMV is managed in HSCT patients, but experience sharing highlighted that not all delegates have access to this therapy. Chieh‐Lin Jerry Teng described how low‐dose valganciclovir provides a valuable option in these situations. Historically Chieh‐Lin Jerry Teng's center in Taiwan managed CMV pre‐emptively in HSCT recipients using antiviral therapy for 2–3 weeks when CMV viremia was detected. Although usually effective, antivirals such as ganciclovir can be associated with hematological toxicities, and therefore, close monitoring is required.^12^ More recently, a prophylaxis approach using low‐dose valganciclovir has been adopted in the clinic and has been associated with a reduction in incidence of CMV infection from approximately 50% of patients to 15% of patients.^26^ Chieh‐Lin Jerry Teng concluded that there may be a role for prophylaxis with low‐dose valganciclovir, especially when letermovir is not available, given the significant reduction in CMV infection. Camille Kotton highlighted that there may be concern with low‐dose valganciclovir increasing the risk of resistant/refractory CMV infections, and this still needs further investigation.

As we learn more about CMV in the HSCT setting, a significant number of unanswered questions and barriers remain. Per Ljungman focused on one of the questions that he believes still needs to be addressed: how can HSCT recipients with repeated CMV reactivations, graft‐versus‐host disease (GvHD) or frequent drug toxicity (“challenging patients”) be managed? He explained that he sees only two or three patients per year in this category, with the management of most patients being possible with standard drugs. For patients he sees with challenging ongoing unmet needs, CMV is often accompanied with poor T‐cell control of CMV, emergence of resistant strains and an increased risk of mortality. For this small group, a number of therapies may be required. Ultimately all CMV management decisions are based on several factors individual to the patient including transplant history (e.g., serology of the donor and recipient, conditioning, and age), GvHD, immune reconstitution, tolerability to drugs, development of antiviral resistance and the option to use immune therapy and, therefore, one size does not fit all.

### 2.3 Understanding CMV risk in the SOT setting

Higher mortality rates have been reported in SOT recipients with CMV compared with uninfected individuals.^12^ However, studies have not yet been able to show that CMV prevention always reduces mortality rates.^32^ Nassim Kamar explained that this is perhaps not surprising given the many confounding factors that place patients in the SOT setting at an increased risk of mortality. It is unlikely that CMV alone can explain the reduced survival rates. As well as causing direct effects (i.e., CMV syndrome and organ invasive disease), CMV infection results in numerous indirect effects, with the total morbidity caused by the indirect effects possibly exceeding that attributed to end organ disease.^1^ Hans Hirsch described that although direct and indirect effects are usually considered separately this may be oversimplifying the situation as there is likely to be some overlap. Data from L'Huillier et al. show that patients who ultimately develop CMV infection after SOT have blunted inflammatory responses before CMV viremia is detected and therefore may have an underlying risk of developing effects usually considered to be indirect; however, it is currently unclear if this is an inherent characteristic of the patient or related to differing medication profiles.^7^ CMV has also been shown to directly modulate both immunosuppressive and immunostimulatory monocyte phenotypes, which could explain some of the indirect effects seen.^33^

Using the heart transplant setting as an example, Nassim Kamar described how organ recipients with CMV infection had increased severity of graft atherosclerosis compared with patients without CMV infection.^34^ Importantly, risk of coronary artery disease, as well as intimal thickening, can be prevented with effective CMV prophylaxis.^35^ With the introduction of more effective prophylaxis agents, a recent study concluded that there is now no link between CMV infection and development of cardiac allograft vasculopathy in heart transplant recipients, which demonstrates the benefits associated with contemporary immunosuppressive and antiviral prophylactic regimens.^36^

It is also important to consider the risk of organ rejection as CMV may be associated with allograft loss.^37^, ^38^ Although some data indicate that graft survival is improved with prophylaxis in kidney transplant recipients^39^, ^40^ the same conclusion has not been associated with pre‐emptive therapy.^32^

Fausto Baldanti reminded delegates that the individuals most at risk of negative outcomes from CMV in the SOT setting were those who were highly immunocompromised.^41^ Fausto Baldanti described experience from his clinic showing that low IE1‐specific T‐cell response in CMV‐seropositive patients prior to kidney transplant predicts susceptibility to CMV complications and subsequent treatment post‐transplant (unpublished data), demonstrating that poor CMV immunocompetence prior to transplant is sustained following surgery in this setting. As a result, the immune‐competence profile of SOT patients prior to surgery should also be considered in determining risk of CMV disease post‐transplant.

### 2.4 Management of CMV in the SOT setting

Camille Kotton discussed the importance of CMV prevention as a key strategy to promote good outcomes following SOT. Post‐transplant prophylaxis or pre‐emptive monitoring is key to preventing CMV, as reflected in the Third International Consensus Guidelines on the Management of Cytomegalovirus in Solid‐organ Transplantation.^12^ A hybrid strategy of surveillance after prophylaxis, using weekly CMV viral load monitoring to detect infection following the initial prophylaxis period, may be of particular importance and utility in high‐risk SOT patients. Although this approach is adopted by experts, it is not strongly evidence based as reflected by the low recommendation and level of evidence grades in the guidelines.^12^ The approach and duration of CMV prevention varies by organ type and the serostatus of both donor and recipient as summarized in the guidelines.^12^ It is also important to consider assessing renal function before initiating therapy and to adjust ganciclovir and valganciclovir doses accordingly. Camille Kotton explained that she provides valganciclovir prophylaxis for 3–6 months, without pre‐emptive monitoring in most individuals except for those at high risk for infection (Patient case 1).

**PATIENT CASE 1:** Low‐level CMV DNAaemia in SOT. When to start treatment?

**Patient**	Heart transplant conducted in 2017 *CMV serostatus of donor*: positive *CMV serostatus of recipient*: negative
**Initial management**	*Induction*: basiliximab/steroids *Prophylaxis*: valganciclovir for 6 months
**Pathway**	*5 months after stopping prophylaxis*: very high viral load; low absolute lymphocyte count. **Valganciclovir** treatment initiated and stopped when CMV DNA was undetectable *<2 weeks after stopping treatment*: CMV DNA 208 IU/mL; absolute lymphocyte count 410 IU/mL (very low). *Next 7 months*: patient monitored regularly and CMV viral load was generally negative so patient was considered to be ‘fine’. *2 years post‐transplant*: several lab tests had been missed. High CMV viral load recorded in April 2019. **Valganciclovir** was reinitiated and continued until CMV DNA was undetectable. *2 weeks after stopping treatment*: CMV DNA 256 IU/mL which increased over next 2 weeks to 3360 IU/ml so **valganciclovir** treatment was restarted. *∼3.5 years post‐transplant*: CMV DNA negative; absolute lymphocyte count increased to > 1600 x 10^3^ /μL.
**Context**	75% of delegates felt management of patients with a high viral load and low lymphocyte count was a challenge with ∼30% of delegates advising that they were seeing more of these patients in the clinic. Most delegates (60–70%) agreed with the presenter that monitoring when low‐level viral load initially increased was appropriate. However, given the very low absolute lymphocyte count, approximately 20% of delegates would have considered treatment at this point – earlier than treatment was initiated in the case. When CMV DNAaemia continued to occur, using a longer course of prophylaxis was considered appropriate by 20% of delegates – an approach ultimately adopted in the case although not recommended in the guidelines due to the increased risk of resistance.
**Conclusion**	CMV infection/relapse correlates with ‘net state of immunosuppression’ and low lymphocyte count.^74^ Low‐level viraemia can suggest CMV infection or just CMV DNAaemia blips. To predict outcome, the viral load should be monitored to see if a trend in increased level is observed.

Case provided by Camille N Kotton.

Abbreviation: CMV, cytomegalovirus.

Camille Kotton acknowledged that there are challenges with the current guidelines and recommended standards of care in the SOT setting. It can be challenging to optimize the duration of prophylaxis to minimize risk of active infection on a case‐by‐case basis while also considering the potential for drug toxicity, the cost of treatment and the challenges around prophylaxis following the development of ganciclovir resistance. Changes to guidelines are anticipated now that trials of letermovir and maribavir in the SOT setting (and brincidofovir in HSCT) have either been published or are in progress.^42^, ^43^, ^44^

Camille Kotton concluded that future guidelines for the management of CMV in SOT should emphasize the importance of infection prevention with effective antiviral therapies, the need for vaccines that are able to induce long‐term immunity, the use of molecular diagnostic tests with a high degree of cross comparability, and the need to optimize the management of resistant/refractory CMV infection after SOT.

## 3 UNDERSTANDING THE CHALLENGE OF CMV RESISTANCE

Use of antiviral agents in both HSCT and SOT settings is associated with a risk of antiviral resistance in immunocompromised patients,^12^, ^45^ a topic discussed by Sophie Alain. Development of CMV resistance can have a negative impact on both graft and patient survival and has been reported to occur in 5%−12% of SOT recipients after ganciclovir therapy.^12^ Resistance prevalence in HSCT is lower (1%−5%) than in SOT recipients, and it is too early to determine if letermovir prophylaxis has influenced rates.^45^

Sophie Alain outlined a three‐step approach to managing CMV resistance. Firstly, and most importantly, patients at risk must be identified. These patients are often those with a prolonged or inadequate exposure to treatment.^46^ Secondly, it is necessary to determine if a resistance mutation is present via genotyping by full‐length Sanger sequencing of target genes.^47^ Identification of known mutations will help assess response to therapy and thus guide treatment pathways on an individualized basis. The third step is to examine treatment options, including new therapeutic options.

Many of the available antiviral agents (e.g., cidofovir, foscarnet, ganciclovir, valganciclovir) and agents in development (e.g., brincidofovir) target viral polymerase pUL54 and are subject to possible cross‐resistance and toxicity.^48^ The need for agents targeting other replication steps has resulted in identification of maribavir that acts on the UL97 viral kinase and letermovir that acts on terminase proteins. In the SOLSTICE trial, maribavir resulted in higher rates of CMV clearance in HSCT and SOT recipients with refractory/resistant CMV than therapy with polymerase inhibitors.^5^, ^49^ Letermovir also shows promise as a salvage therapy, but resistance can develop rapidly, especially if patient compliance is poor or treatment is interrupted.^50^

Although therapeutic options are expanding with the introduction of antivirals without cross‐resistance, Sophie Alain concluded that the burden of refractory CMV infections and resistance will remain an unmet need until more evidence from randomized clinical trials is available.

### 3.1 Immunotherapy in the management of CMV in transplant settings

Humoral, cellular, innate, and adaptive immune responses are all involved in the response to CMV in order to limit viral replication and achieve viral latency. Sustained control of CMV infection is largely due to cellular immunity with a diverse T‐cell response occurring that is targeted toward CMV‐specific CD4+ and CD8+ cells.^51^ In the first days after SOT, there is a marked decrease in CD8+ T cells and immunoglobulin G (IgG) levels so that immunity against CMV is reduced. However, the immune response varies depending on the organ being transplanted and the CMV status of the donor and recipient. Marty Zamora explained that transcriptional profiling of peripheral blood from lung transplant recipients revealed that many immune‐related genes (for example, genes encoding interferon [IFN]‐inducible factors and chemokines) are upregulated in CMV‐infected patients compared with non‐infected patients.^52^ This puts patients at an increased risk of CMV infection and highlights the need for prophylaxis.

The potential reduction in immune control of CMV post‐transplant suggests a role for CMV immunoglobulin (CMVIG) preparations in SOT recipients. Pooled plasma derived from donors with high CMV antibody titers provides a source of CMV‐specific polyclonal immunoglobulin.^53^ Two CMVIG products are currently available that have similar CMVIG antibody concentrations and neutralization titers. Marty Zamora explained that CMVIG influences the immune response by multiple mechanisms including virus neutralization by anti‐CMV, an effect on maturation of dendritic cells, decreased T‐cell activation and decreased cytokine production.^51^

A number of potential uses of CMVIG have evolved over time. Piedad Ussetti described the promising results obtained in her center in Madrid, with the current protocol for minimizing risk of CMV disease in seronegative lung transplant recipients with the combination of 12 months of valganciclovir and CMVIG administered at weekly intervals for the first month and then monthly for the first year (unpublished data). In the heart transplant setting, Udo Boeken described the CMV prophylaxis regimen in heart transplant recipients adopted by his center at the Heinrich Heine University in Düsseldorf. Ganciclovir/valganciclovir is administered for 90 days post‐surgery to most individuals; the exception being seronegative recipients receiving an organ from a seronegative donor (D‐/R‐).^54^ For those considered at highest risk of disease (D+/R‐), 3 days of CMVIG treatment is added to the management pathway. Application of this protocol has resulted in survival rates 1‐year post heart transplant of 77.8% in D+/R‐, 74.4% in D‐/R+, 87.5% in D‐/R‐, and 71.7% in D+/R+.

There is evidence suggesting that CMVIG, either alone or in combination with antivirals, may improve overall survival and decreases CMV disease in SOT recipients,^54^, ^55^, ^56^ but further robust trials are required to identify its optimal role in the management of CMV (Patient case 2).

**PATIENT CASE 2:** The role of CMVIG in managing severe disease in the SOT setting

**Patient**	34‐year‐old heart transplant recipient *CMV serostatus of donor*: positive *CMV serostatus of recipient*: negative. Pre‐transplant IgG anti‐CMV levels were <20 U/mL. IFNγ from CD8 response to IE1 was present (2.75%)
**Initial management**	Ganciclovir for 15 days followed by valganciclovir
**Pathway**	*Day 7*: patient symptomatic (clostridium difficile‐associated diarrhoea, pneumonia) *Day 30*: patient symptomatic *(clostridium difficile‐associated diarrhoea, pneumonia)* and CMV disease (CMV DNA >101,300 IU/mL). **Ganciclovir** restarted for 7 days. *Day 37*: Viral load remained high (63,100 IU/mL). IgG levels tested and severe HGG diagnosed (3.32 g/L). **CMVIG** administered (150 mg/kg followed by six doses of 100 mg/kg until DNAemia negative. *Day 90*: IgG 1090 mg/dL; *Day 120*: IgG 767 mg/dL
**Context**	63% of delegates would have tested IgG levels in an SOT recipient with difficult‐to‐control CMV disease as represented in this case Most (69%) had a protocol in place for IgG replacement
**Conclusion**	After heart transplantation, many patients will have broad immune deficiency and the immunological status of the patient is likely to contribute to the difficulty in controlling any subsequent CMV disease. When considering use of CMVIG, lack of CMV‐specific T cells, presence of HGG, CD4+/CD8+ or NK lymphocytopenia could all suggest high risk of disease. The tools to assess these factors are accessible in most clinics based on feedback provided by delegates during the case discussion.

Case provided by Javier Carbone.

Abbreviations: CMV, cytomegalovirus; CMVIG, cytomegalovirus immunoglobin; HGG, hypogammaglobulinaemia; NK, natural killer cell.

In the HSCT setting, a role for CMVIG also still needs to be established. Approximately one‐third of HSCT recipients will experience reactivation after the end of letermovir prophylaxis,^27^ it is possible that the use of CMVIG in these patients may be appropriate in a properly designed clinical trial.

## 4 MANAGEMENT OF CMV INFECTION IN PEDIATRIC SOT PATIENTS

CMV infection and disease are common in pediatric SOT recipients. Lara Danziger‐Isakov explained that the incidence varies depending on the type of organ transplant, but CMV infection rates of 40% in pediatric liver transplant recipients and 15% of pediatric kidney transplant recipients have been reported.^12^ Available data suggest that CMV infection/disease in pediatric SOT patients could be associated with adverse outcomes, including organ rejection and CMV DNAemia blips, but evidence is more limited than in adult patients.

Prophylaxis with intravenous ganciclovir or oral valganciclovir is the standard CMV prevention strategy for pediatric SOT patients, with some high‐risk groups receiving more targeted prophylaxis (e.g., adjuvant CMVIG) and increased surveillance testing.^12^, ^57^ However, breakthrough CMV DNAemia during prophylaxis treatment is common in children.^58^ Lara Danziger‐Isakov explained that calculating the dose of antiviral medication to use is a challenge in the pediatric setting.^12^ Other areas that need further refinement and research include the risk of late‐onset CMV disease and alternative treatment options in the case of antiviral resistance. Inducing CMV‐specific immune responses in children, for example using CMV‐specific T cells, was seen to be an interesting approach by Lara Danziger‐Isakov, but the impact of these strategies in young children with developing immune systems requires further research.

## 5 TOWARD PERSONALIZED MANAGEMENT—USING CMV‐SPECIFIC IMMUNITY TO INFORM DECISION MAKING

Glen Westall discussed how transplantation provides an ideal set of circumstances for CMV to take hold, with the immune system suppressed and CMV entry disguised within a major MHC‐mismatched allograft. The presentation of CMV peptides on host/self MHC molecules is required for T cells to recognize virally infected cells; however, in MHC‐mismatched allografts, T cells may not be able to recognize the CMV peptides being presented. Glen Westall explained that this is likely to be most important in lung and liver transplant settings where there is a high CMV burden. Furthermore, in the setting of lung transplantation, natural killer cells and γδ T cells may also be important for controlling CMV than conventional T cells as CMV replication in lung allografts has been associated with the enrichment of γδ T cells rather than conventional T cells.^59^

Glen Westall discussed the predictive ability of CMV immunoassays. These assays might predict CMV risk in some patients, but not necessarily in all. Despite this, even an indeterminate result can be useful as it suggests a high degree of immunosuppression and thus a higher risk of CMV. Glen Westall suggested that in donor‐positive/recipient‐negative situations, CMV infection is effectively suppressed, while the patient is on prophylaxis and, therefore, immunity does not develop. However, following discontinuation of prophylaxis, CMV infection may occur and immunity can develop. Consequently, there is limited utility for assessing CMV immunity in this group of patients while they remain on prophylaxis. Where CMV immunity assays may have value is in determining the optimal length of prophylaxis for each patient (especially in those seropositive prior to transplant) and the need for secondary prophylaxis.^60^

Glen Westall concluded that assays for CMV immunity are a useful addition to the diagnostic toolkit, but optimal thresholds for immune markers to accurately predict risk, manage patient outcomes, and inform the need for prophylaxis are still required (Patient case 3).

**PATIENT CASE 3:** Difficult‐to‐treat CMV infection after lung transplantation

**Patient**	26‐year‐old with cystic fibrosis Bilateral lung transplant in March 2014 *CMV serostatus of donor*: positive *CMV serostatus of recipient*: negative
**Initial management**	*Induction*: basiliximab *Immunosuppression*: prednisone, tacrolimus, mycophenolate mofetil *Prophylaxis*: intravenous **ganciclovir** for 7 days; intravenous **CMVIG** and oral **valganciclovir** for 12 months
**Pathway**	*Year 1 post‐transplant*: exponential increase in viraemia (>40,000 IU/mL). Switched to everolimus and tacrolimus dose decreased.*Assessments*: *CMI (by intracellular cytokine staining*: 12 CMV‐specific CD8+ T cells/μL; 9 CMV‐specific CD4+ T cells/μL *UL97 genotyping*: C592G (low‐level ganciclovir resistance); L595S (high‐level ganciclovir resistance). *Follow‐up*: high‐dose **ganciclovir** initiated. Viral load declined to undetectable levels but neutropenia developed. **Foscarnet** initiated. Hypomagnesaemia developed and viral load increased. **Leflunomide** initiated. Viraemia was controlled. Patient has no ongoing CMV disease.
**Context**	Extending valganciclovir prophylaxis beyond 3–6 months is the best approach to prevent CMV infection and disease in the lung transplant setting. Risk of bronchiolitis obliterans syndrome and poor graft outcomes post‐lung transplantation support the control of viraemia in recipients even in the absence of symptoms Despite identification of variants associated with ganciclovir resistance, low‐level resistance has been shown to be overcome by higher doses of ganciclovir Letermovir and maribavir were not available at this time, so management options shown may be different in the current era
**Conclusion**	In this case, being able to use the tools available to identify exactly what challenges had to be overcome (including the monitoring for CMV‐specific CMI) resulted in a good outcome. However, the case demonstrates that management may need to evolve in order to address the challenges as they arise.

Case provided by Mario Fernández‐Ruiz.

Abbreviations: CMI, cell‐mediated immunity; CMV, cytomegalovirus; CMVIG, cytomegalovirus immunoglobin.

## 6 LOOKING TO THE FUTURE OF CMV MANAGEMENT IN THE TRANSPLANT SETTING

### 6.1 Novel therapeutics

Dana Wolf discussed the considerable need for anti‐CMV agents with alternative modes of action to those currently available. The antimalarial artemisinin derivative, artesunate, inhibits CMV via the inhibition of the host‐cell functions required for virus replication.^61^ However, studies have demonstrated the highly divergent nature of this drug and therefore more potent artemisinin derivatives such as artemisone are currently being investigated. Artemisone has similar in vitro efficacy to ganciclovir but acts at an earlier stage of the CMV replication cycle. It has synergistic antiviral activity in combination with approved and experimental anti‐CMV drugs, with the highest effect demonstrated with the viral UL97 kinase inhibitor, maribavir.^62^

Dana Wolf explained that a major limitation in CMV translational research has been the lack of representation of the viral natural tropism in in vitro cell‐type cultures. However, nasal turbinate organ culture is now being used to study the earliest steps of infection and the subsequent immune response.^63^ This should start to more clearly elucidate the initial events occurring after CMV infection and provide a model for assessing new antiviral interventions.

Dana Wolf concluded by emphasizing that mechanism‐informed drug repurposing and advanced ex vivo human models of CMV infection have shown great advances in tackling tissue innate immune responses and new therapies for CMV.

### 6.2 Novel cellular therapies

Cellular therapy for CMV in the HSCT field started 30 years ago,^64^ but these therapies are still not being used in routine clinical practice; a situation highlighted by Patrizia Comoli. Cellular therapy triggers the secondary immune response to viruses by stimulating donor cells from HSCT recipients in vitro using infected cells, antigen‐presenting cells (APC) rich in specific peptides, or APCs transduced with vectors carrying relevant antigen products.^65^ Polyclonal populations of virus‐specific T cells (VST) are generated that produce high levels of IFNγ, and other cytokines in a process can take between 2 and 8 weeks depending on the protocol used. Cellular infusions as prophylaxis can prevent CMV infection in HSCT recipients, while treatment of infected individuals results in a clearance rate of 82%. The large number of cells generated means that this method is suitable for patients requiring repeated infusions.

Patrizia Comoli highlighted that some patients need more rapid treatment with CD8+ cells to clear infection quickly and explained that the process to develop cellular infusions can be accelerated using magnetic beads coated with anti‐IFNγ to select CD4+/CD8+ cells and using beads with a streptamer tag to select CD8+ T cells.^65^, ^66^ The efficacy observed with these protocols is similar to that seen with cultured cells.

In the SOT setting, autologous VSTs have been shown to be effective even in patients using steroids or immunosuppressants, although more information is required on the potential risk of late rejection in these patients.^67^ One factor that may have led to the reluctance in adoption of cellular therapy is the theoretical risk of toxicity; however, Patrizia Comoli explained that this has not been observed in the clinic. Lack of access to therapy may also contribute to the slow uptake, but Patrizia Comoli explained that introduction of third‐party VSTs, especially those optimized based on HLA typing and specific function, had started to address this limitation. Patrizia Comoli left delegates with the thought that it is now time to move the field forward by conducting well‐controlled studies to further elucidate the potential for cellular therapies after transplant (Patient case 4).

**PATIENT CASE 4:** Cellular therapy in CMV reactivation after HSCT

**Patient**	52‐year‐old with acute myeloid leukaemia HSCT with a haploidentical donor *CMV serostatus of donor*: negative *CMV serostatus of recipient*: positive
**Initial management**	*Antiviral therapy*: **letermovir** up to Day 100
**Pathway**	*Day 100*: complete remission and no GvHD. *Next 2 months*: platelet and neutrophil counts declined. *Day 180*: CMV and parvoB19 reactivation detect. Graft function poor. *Management*: High‐dose **CMVIG**, **foscarnet**, **boost of CD34+ cells** (same donor). Second course of **CMVIG** and **foscarnet**. *Outcome*: CMV and parvoB19 viraemia cleared. Second course of **letermovir** with no subsequent reactivation.
**Context**	CMV reactivation occurs after Day 100 in approximately 10% of patients. However, use of letermovir after Day 100 is off‐label. If this approach is being considered it may be best to wait for viraemia to clear to minimise risk of resistance
**Conclusion**	Combining cellular therapy with CMVIG and antiviral therapy resulted in good outcomes for this patient; however, specific protocols are currently not available to guide patient management.

Case provided by Fabio Ciceri.

Abbreviations: CMV, cytomegalovirus; CMVIG, cytomegalovirus immunoglobulin; GvHD, graft‐versus‐host disease; HSCT, haematopoietic stem cell transplant.

### 6.3 Vaccines

Antibody responses induced by vaccination have the potential to reduce the high levels of viremia associated with CMV disease and limit the quantity of virus transferred from donor to recipient in the SOT setting.^68^, ^69^ Paul Griffiths described how vaccination with recombinant glycoprotein B (gB) plus an MF59 adjuvant increased anti‐gB titers in both seronegative and seropositive individuals awaiting SOT.^68^ It also reduced viral load parameters after transplantation and he suggested that could be a result of reduced transmission of virus from the donated organ to the recipient.

The human CMV gB protein has five antigenic domains (ADs), with AD1 being immunodominant.^70^ In vaccinated seronegative individuals in an SOT setting, vaccination does not appear to have a direct effect on levels of neutralizing antibodies; instead, transplant acts as a prime boost to subsequently increase neutralizing antibody titers.^71^ In seropositive individuals, vaccination can boost all ADs, but only AD2 correlates with protection.^72^ Paul Griffiths explained how a future study infusing antibodies directed toward the CMV gB AD2 into a seronegative recipient, and then transplanting an organ from a seropositive donor, could allow the quantity and quality of antibodies required to elicit protection to be determined.

Two further vaccines are being studied: a non‐replicating viral vector platform based on lymphocytic choriomeningitis virus, with the authentic glycoprotein replaced with gB from CMV, has been shown to have a good safety profile in preclinical studies, with high levels of neutralizing antibodies and cell‐mediated immunity induced,^73^ and rdCMV/DISC (V160) is a whole virus construct that has also been reported to increase cellular and humoral immunity.^69^

Paul Griffiths concluded that he hoped that results from the studies investigating the safety and efficacy of the different vaccines would encourage manufacturers to initiate the Phase 3 studies required to push the field forward.

## 7 CONCLUSION

During the well‐attended symposium, the major advances in the CMV field were presented and discussed. As understanding of the immune effects of CMV is continuing to increase, Atul Humar concluded the meeting by suggesting that it is the start of a new era in management of CMV. He was optimistic that, once thresholds for CMI had been established, CMV risk would be better understood in both HSCT and SOT, leading to improved patient management. Viable alternatives to antiviral therapy, such as cellular or immuno therapies, are becoming established, and, as more data become available, it is likely that management protocols will evolve to become more individualized based on the patient's immune footprint. The new era in CMV is likely to feature multiple diagnostic, therapeutic, and preventive strategies that promise to change the paradigm of CMV.

## CONFLICT OF INTEREST

In addition to speaking at the Biotest AG‐sponsored International Symposium, the following disclosure/conflict of interest statements are provided: *Camille N. Kotton*: Member of the Advisory Committee on Immunization Practice (ACIP) at the US Centers for Disease Control and Prevention. Consultant for Biotest, Evrys, ExeVir, Merck, Takeda, Hookipa and Oxford Immunotec. Recipient of grant from NIH. *Julián Torre‐Cisneros*: Recipient of research grants from Cellestis (a QUIAGEN company), MSD and Roche. Recipient of educational grants from MSD and Roche. Member of advisory boards for Biotest, MSD and Roche. The research has been supported by CIBER ‐ Consorcio Centro de Investigación Biomédica en Red‐ (CB 2021), Instituto de Salud Carlos III, Ministerio de Ciencia e Innovación and Unión Europea—NextGenerationEU.

### The international CMV symposium faculty

*José Maria Aguado*: Honoraria for speaking or participating in Advisory Boards and/or research grants from Biotest, Gilead Sciences, MSD, Pfizer, Roche. *Sophie Alain*: Research funding as a scientific expert or site principal investigator from Altona, Biotest, BioMérieux, Qiagen, Hologic, GlaxoSmithKline, Merck, MSD, Shire (a Takeda Company). Scientific expert/Advisory board member for the QCMD. ANSM expert for delivery of anti‐CMV for compassionate use in refractory/resistant patients. *Fausto Baldanti*: Consultant for: Biotest, Takeda, Qiagen, Roche, MSD; Research support by: DiaSorin, NTP, Qiagen, Elitech. *Udo Boeken*: Consultant and speaker for Biotest. *Javier Carbone*: Speaker/Advisory Board member for Biotest, Grifols and LFB. Research funding from Biotest, Grifols and Takeda. *Patrizia Comoli*: Speaker/Advisory Board member for Atara Biotherapeutics and Pierre Fabre Pharma. *Lionel Couzi*: Grants from Astellas and Novartis. Lecture fees from Astellas, Biotest, Chiesi, GSK, Novartis, Ostuka, Sandoz. Consultancy fees from Biotest, Hansa and Novartis. Travel funding from Astellas, Chiesi, Novartis, Sandoz and Vifor. *Lara Danziger‐Isakov*: Consultant for Merck and Takeda and contracted clinical research with Ansun BioPharma, Astellas, Merck, Pfizer, Viracor and Takeda. Recipient of grant from NIH. *Corrado Girmenia*: Speaker/Advisory Board member for AbbVie, Amgen, Biotest, Gilead Sciences, Janssen, MSD, Novartis Pharma, Sanofi and Takeda. *Paolo Grossi*: Advisory Board member for Allovir, Biotest, Gilead Sciences, MSD and Takeda. Speaker's bureau for Atara, Biotest, Gilead Sciences, MSD, Shionogi and Takeda. *Hans H. Hirsch*: Consultant honoraria from Molecular Probes and Roche Diagnostics and Speaker honoraria from Gilead Sciences. *Atul Humar*: Research support from Astellas, Roche, Qiagen. Advisory Board member: Merck, Takeda. *Nassim Kamar*: Speaker/Advisory Board member for AbbVie, Astellas, Astrazeneca, Biotest, CSL Behring, Chiesi, Gilead Sciences, Fresenius Medical Care, MSD, Neovvi, Novartis Pharma, Sanofi, Sandoz, Shire (a Takeda company). *Per Ljungman*: Advisory board/endpoint committee member for AiCuris, Merck, MSD, Takeda. Investigator with MSD. Speaker for Biotest, MSD, Takeda. *Michele Malagola*: Advisory Board member for Biotest and MSD. *Nicolas Mueller*: Advisory Board member for MSD, Pfizer and Takeda. *Martina Sester*: Research support/honoraria and travel support from Astellas, Biotest and Novartis. *Glen Westall*: Advisory Board member for CSL Behring and research study support from Qiagen. *Dana Wolf*: Speaker/Advisory Board member for Merck, MSD and Takeda. Recipient of research grants from GSK, MSD, and Sanofi. *Marty Zamora*: Consultant for CSL Behring.

## Supporting information

Graphical Abstract
