# Supplementary material for: Cytomegalovirus in the transplant setting: Where are we now and what happens next? A report from the International CMV Symposium 2021
Source: Transpl Infect Dis. 2022 Nov 11;24(6):e13977. doi: 10.1111/tid.13977 (PMC10078482; doi:10.1111/tid.13977)
Supplement: Supplementary file 1 — Graphical Abstract [file TID-24-0-s001.pptx]

## Slide 1
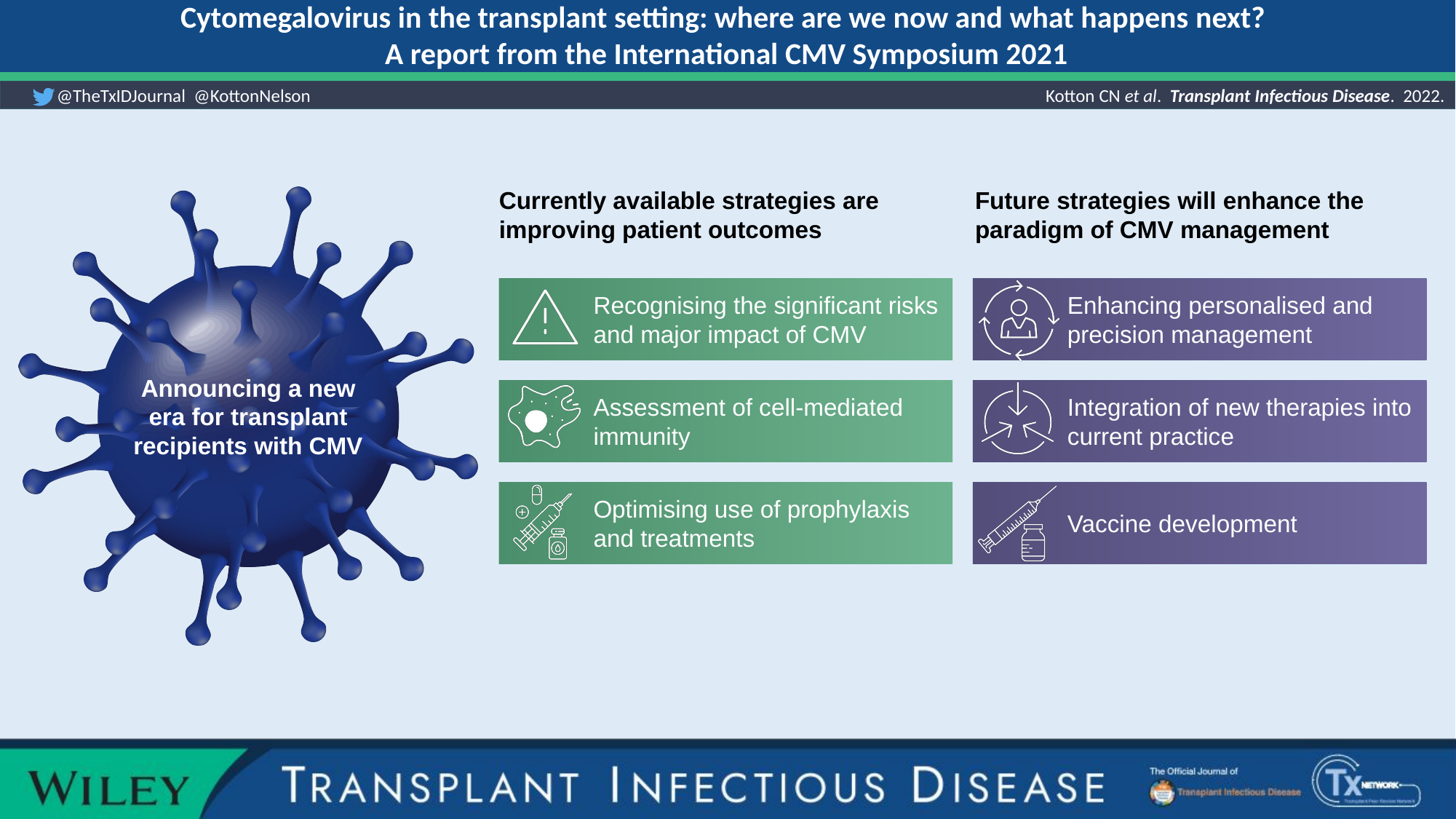

Cytomegalovirus in the transplant setting: where are we now and what happens next?
A report from the International CMV Symposium 2021
Kotton CN et al. Transplant Infectious Disease. 2022.
 @TheTxIDJournal @KottonNelson
Currently available strategies are improving patient outcomes
Future strategies will enhance the paradigm of CMV management
Announcing a new era for transplant recipients with CMV
Recognising the significant risks and major impact of CMV
Enhancing personalised and precision management
Integration of new therapies into current practice
Assessment of cell-mediated immunity
Optimising use of prophylaxis and treatments
Vaccine development
